# *In vitro* RNA interference targeting the DNA polymerase gene inhibits orf virus replication in primary ovine fetal turbinate cells

**DOI:** 10.1007/s00705-013-1896-z

**Published:** 2013-11-01

**Authors:** Gaili Wang, Wenqi He, Deguang Song, Jida Li, Yingfu Bao, Rongguang Lu, Jingying Bi, Kui Zhao, Feng Gao

**Affiliations:** 1 Key Laboratory of Zoonosis, Ministry of Education, College of Veterinary Medicine, Jilin University, 5333 Xi’an Road, Changchun, 130062 China; 2 Key Laboratory of Zoonosis, Ministry of Education, Institute of Zoonosis, Jilin University, 5333 Xi’an Road, Changchun, 130062 China

## Abstract

Orf, which is caused by orf virus (ORFV), is distributed worldwide and is endemic in most sheep- and/or goat-raising countries. RNA interference (RNAi) pathways have emerged as important regulators of virus-host cell interactions. In this study, the specific effect of RNAi on the replication of ORFV was explored. The application of RNA interference (RNAi) inhibited the replication of ORFV in cell culture by targeting the ORF025 gene of ORFV, which encodes the viral polymerase. Three small interfering RNA (siRNA) (named siRNA704, siRNA1017 and siRNA1388) were prepared by *in vitro* transcription. The siRNAs were evaluated for antiviral activity against the ORFV Jilin isolate by the observation of cytopathic effects (CPE), virus titration, and real-time PCR. After 48 h of infection, siRNA704, siRNA1017 and siRNA1388 reduced virus titers by 59- to 199-fold and reduced the level of viral replication by 73-89 %. These results suggest that these three siRNAs can efficiently inhibit ORFV genome replication and infectious virus production. RNAi targeting of the DNA polymerase gene is therefore potentially useful for studying the replication of ORFV and may have potential therapeutic applications.

## Introduction

Orf, also known as contagious ecthyma or cutaneous pustular dermatitis, is an acute contagious disease of sheep, goats, wild ruminants, and humans worldwide. It is caused by orf virus (ORFV), which is an epitheliotropic parapoxvirus, the prototype member of the genus *Parapoxvirus*, family *Poxviridae* [1]. ORFV generally induces proliferative and self-limiting lesions around the mouth, teats and skin of affected animals, especially in young lambs [2].The mortality rate is as high as 93 % in kids when lesions of the lips and udders prevent them from receiving nutrition, thus leading to rapid emaciation [3, 4]. Outbreaks of orf have occurred in many countries where substantial numbers of sheep and goats are raised [5]. The disease not only causes huge economic losses to the sheep industry but also, as a zoonotic disease, poses a threat to human health [2].

The ORFV genome is an approximately 140-kbp double-stranded DNA containing at least 132 putative genes [6]. Like other poxviruses, the ORFV genome contains a large central coding region (ORFs 009 to 111), which includes homologues of conserved poxvirus genes involved in basic replicative mechanisms and structure and morphogenesis of intracellular mature and extracellular enveloped virions, bounded by two identical inverted terminal repeat regions [7]. ORF025 encodes the DNA polymerase (DNA Pol), which is the core enzyme in the process of ORFV replication and catalyzes the replication of the viral genome.

RNA interference (RNAi) is a conserved gene-silencing mechanism that is induced by 19- to 27-nucleotide (nt) small interfering RNA (siRNA) molecules that are homologous to the target genes [8, 9]. RNAi technology is not only a powerful tool for functional genomics studies but is also a potentially useful antiviral method, and it is increasingly being used to inhibit the replication of viral pathogens [10]. Therefore, it has been suggested that the preferred method for controlling the virus is to interfere with replication of the viral genome and the expression of viral genes. We designed specific siRNAs to target the DNA polymerase genes of ORFV in order to test whether RNAi could selectively target ORFV viral DNAs. This study provides not only an experimental basis for the development of a new anti-ORFV strategy but also a new approach for studying ORFV infection and replication.

## Materials and methods

### Cells and virus propagation

Primary ovine fetal turbinate (OFTu) cells were cultured in minimal essential medium (MEM; (Hyclone) with 10 % fetal bovine serum (FBS; Hyclone), 2 mM L-glutamine, and 100 U of penicillin, 100 μg of streptomycin, and 20 μg of nystatin per ml in a 37 °C, 5 % CO_2_ incubator. Orf virus was isolated from scabs collected from lesions of a 6-week-old small-tailed Han sheep showing typical symptoms of orf virus infection in November 2008 in Jilin Province, China [2]. When 90 % of the virus-infected cells showed a cytopathic effect (CPE), the cultures were collected after undergoing three freeze-thaw cycles.

### Isolation of genomic DNA

DNA was extracted from CPE-positive cell cultures using a Takara MiniBEST Viral DNA Extraction Kit (Takara, Dalian, China) according to the manufacturer’s instructions and was used as template in PCR.

### PCR amplification

The ORF025 gene was amplified by PCR from DNA extracted from CPE-positive cell cultures. The complete ORF025 (DNA polymerase) gene sequence was divided into three sections called “DNA polymerase I”, “DNA polymerase II” and “DNA polymerase III” and specific primers were designed using Primer Premier 5.0 software based on published ORFV (ORFV-OV-SA00 strain) genomic sequences available in the NCBI GenBank database (accession number AY386264.1). These primers were custom synthesized by Shanghai Sangon Biological Engineering Technology and Services Co., Ltd., China. The six primer sets used in this study were specific for ORF025 (Table 1). The PCRs for DNA polymerase I had an initial denaturation step of 95 °C for 2 min, followed by 35 cycles of 94 °C for 45 s, 59 °C for 45 s, 72 °C for 45s, and a final extension of 72 °C for 10 min. The PCR for DNA polymerase II had an initial denaturation step of 95 °C for 2 min followed by 30 cycles of 94 °C for 45 s, 58 °C for 45 s, and 72 °C for 1 min, and a final extension at 72 °C for 10 min. The PCR for DNA polymerase III had an initial denaturation step of 95 °C for 2 min, followed by 30 cycles of 94 °C for 30 s, 55 °C for 30 s, and 72 °C for 150 s, and a final extension at 72 °C for 10 min. PCR was carried out in 25-μL reaction mixtures containing 2.5 μL PCR buffer, 200 mM each dNTP, 0.2 mM each oligonucleotide primer, 100 ng of each DNA sample, 0.5 U rTaq DNA polymerase (Takara, Dalian, China), and 10 μL sterilized water. Amplicons were visualized by electrophoresis in 1 % agarose gels and documented using a gel documentation system.

**Table 1:** Primers used in this study

Name	Primer	Sequence (5’-3’)
DNA polymerase I	FP	TTCCGTCGGATGGGCTGCT
	RP	TCCGTGTTCCTGGAGGTGGG
DNA polymerase II	FP	CGCCTCGAACTCCACCTTG
	RP	GCCTCTACCTCTGGTCGCACT
DNA polymerase III	FP	TGTTGTAGTCGAAGATGA
	RP	GCCGCAGCACGATGAAGAT
B2L	FP	GGGGCGGCGTAT TCTTCT
	RP	GCTGTTCTTGGCGTTCTCG

### Cloning and DNA sequencing

The PCR products of the DNA polymerase I, II, and III fragments were cloned into the pMD-18T vector (Takara, Dalian, China) and used to transform *E. coli* DH5a. At least six positive clones of each selected amplification product were sequenced at Shanghai Sangon Biological Engineering Technology and Services Co., Ltd., China. The three sequences were combined to obtain the complete sequence of ORF025.

### ORFV siRNA preparation

Based on the complete sequences of ORF025, four pairs of siRNAs, D704, D1017, D1388 and negative siRNA control (scrambled siRNA) were designed and synthesized by Shanghai GenePharma Co., Ltd. The sequences are shown in Table 2.

**Table 2:** List of siRNA sequences used in this study

Name		Sequence (5’-3’)
siRNA704	up	GGCUGUUGUAGUCGAAGAUTT
	down	AUCUUCGACUACAACAGCCTT
siRNA1017	up	GCGAGUAGUUUGCGUACAUTT
	down	AUGUACGCAAACUACUCGCTT
siRNA 1388	up	GUCCCGUUGUUGUUGUUGATT
	down	UCAACAACAACAACGGGACTT
Negative control siRNA	up	UUCUCCGAACGUGUCACGUTT
	down	ACGUGACACGUUCGGAGAATT

### Cell culture and transfection

OFTu cells were cultured in 24-well plates at a concentration of 1.0 × 10^5^ cells/well and incubated in a CO_2_ incubator with a 5 % CO_2_ atmosphere. When the cells were 50-60 % confluent, siRNAs were introduced using X-tremeGENE siRNA transfection reagent (Roche, USA) according to the manufacturer’s protocol. Briefly, 2.5 μL of transfection reagent and 0.25 μg of siRNA were added to each well and incubated for 6 h. The cells were then washed with MEM without serum and further cultured for 16 h in MEM supplemented with 2 % fetal bovine serum prior to viral infection. The siRNA-treated cells were infected with 100 TCID_50_ (10^9.0^) of ORFV, and the infection was allowed to proceed for the indicated time periods. Untransfected OFTu cells were used as a control.

### Determination of virus genome copy number

Infected OFTu cells and culture supernatants were collected 48 h after ORFV infection. DNA was extracted from collected CPE-positive cell cultures using a Takara Minibest Viral DNA Extraction Kit (Takara, Dalian, China) according to the manufacturer’s instructions. PCR was used to amplify the B2L gene (GenBank accession no. AY386264.1) with FP and RP primers (designed using Primer Premier 5.0 software) under conditions of 94 °C for 3 min, followed by 40 cycles of 94 °C for 30 s, 60 °C for 30 s, and 72 °C or 60 s, with a final extension at 72 °C for 7 min. The PCR product was gel-purified using an Agarose Gel DNA Extraction Kit (Roche) and then cloned into pMD-18T vector (Promega Corp., Madison, Wisc., USA). The resulting plasmid, pMD-B2L, with the correct sequence confirmed by direct sequencing, was selected as a quantitative standard for determination of the viral DNA copy number. Real-time PCR was performed using a CFX96 PCR Cycler with a Bio Easy SYBR Real-time PCR Kit (Hangzhou Bioer Technology Co., Ltd, China) under the conditions of 96 °C for 2 min 30 s, followed by 40 cycles of 95 °C for 30 s, 60 °C for 30 s, and 72 °C for 30 s, with a final extension at 72 °C for 10 min. A standard curve for the determination of ORFV genome copy numbers was created by real-time PCR of standard plasmid pMT-B2L preparations at serial dilutions of 10^1^, 10^2^, 10^3^, 10^4^, 10^5^, 10^6^ and 10^7^ copies/μL. The specificity of the real-time PCR was confirmed by sequencing the product.

## Results

### PCR amplification of the orf virus DNA polymerase gene

**Fig. 1:**
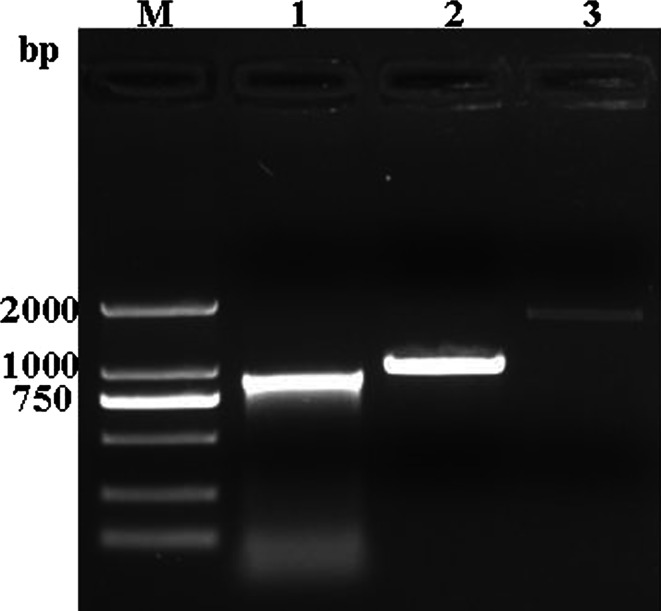
Amplification of the ORFV DNA polymerase gene by PCR. Lane M, DNA marker DL-2000; lane 1, DNA polymerase I PCR product (888 bp); lane 2, DNA polymerase II PCR product (1001 bp); lane 3, polymerase III PCR product (1752 bp)

Specific products of the expected size (888 bp, 1001 bp and 1752 bp; Fig. 1) were obtained when a sample extracted from CPE-positive OFTu cells was amplified by PCR. The nucleotide sequences of these amplicons were combined to obtain the complete sequence of the ORFV 025 gene (3039 bp).

### CPE analysis

**Fig. 2:**
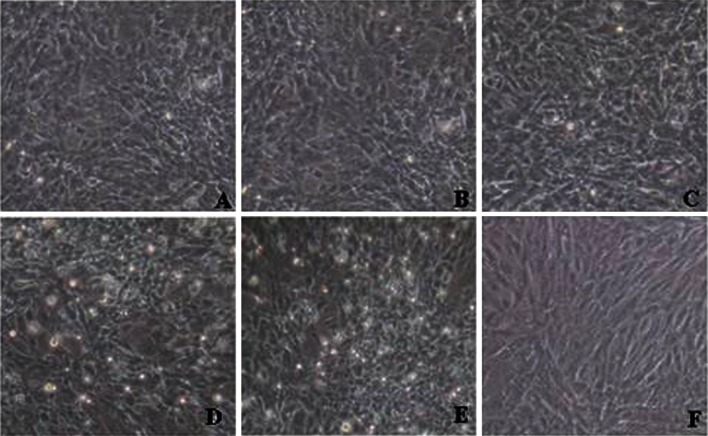
Effect of siRNAs on ORFV-induced CPE in OFTu cells. OFTu cells were transfected with different siRNAs and then infected with ORFV.** A**-**D** Cells were transfected with siRNA704, siRNA1017, siRNA1388, and negative control siRNA, and CPE was examined 48 h postinfection.** E** cells that were infected ORFV at 48 h.** F** Untreated healthy OFTu cells

Transfection complexes (siRNAs and transfection reagent) were completely removed after transfection for 6 h, and 100 TCID_50_ of orf virus suspensions was added to each well. Untransfected cells were used as a control. At 48 h after orf virus infection, observation of CPE indicated siRNA704, siRNA1017 and siRNA1388 appeared to inhibit virus replication to varying degrees (Fig. 2).

### Examination of siRNA effect by infectious virus assay

**Fig. 3:**
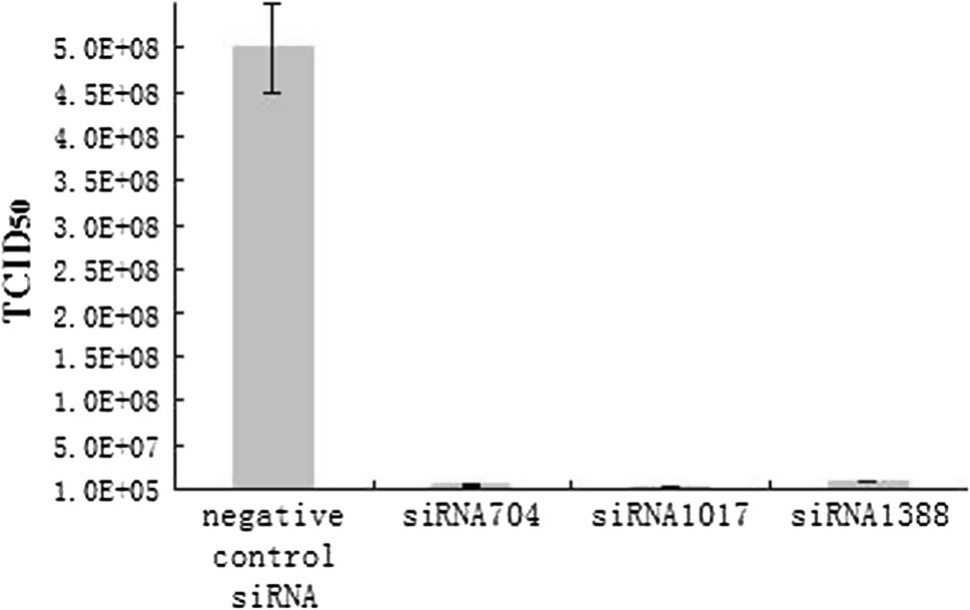
Inhibition of virus production in siRNA-treated cells. TCID_50_ values are the means of three repeat titrations at the time points indicated

To further investigate the inhibitory effect of siRNAs, the microtiter method was used for titration of orf virus at 48h postinfection, and the results (Fig. 3) showed that, in control cells transfected with scrambled siRNA, titers reached 1 × 10^8.7^/0.1 ml (TCID_50_) at 48 h postinfection, similar to mock transfection. In contrast, titers at 48 h postinfection were 1×10^6.67^, 1×10^6.40^ and 1×10^6.93^/0.1 ml for cells transfected with siRNA704, siRNA1017 and siRNA1388, respectively, which corresponded to a 105-,199-, and 59-fold reduction in comparison to cells transfected with scrambled siRNA.

### Examination of siRNA effect by real-time PCR

**Fig. 4:**
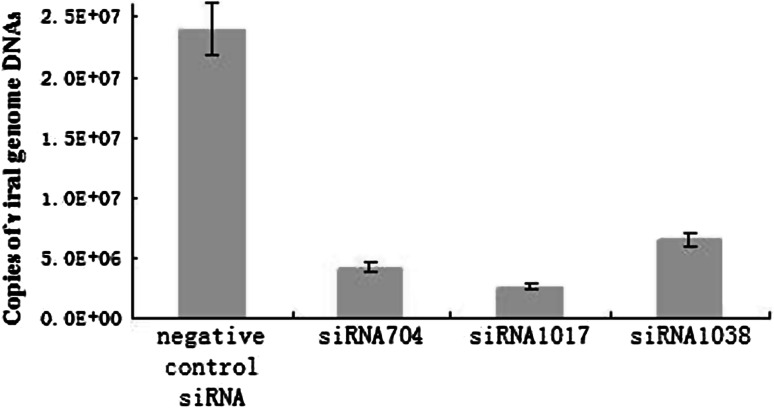
Reduction of viral genome copy number after siRNA treatment in OFTu cells 48 h postinfection. The ORFV genome copy numbers are the means of three repeat experiments

To quantify the effect of siRNA on viral replication, the viral genome copy number was determined at 48 h postinfection by real-time quantitative PCR, using serially diluted plasmid pMD-B2L as a standard. The correlation coefficient (R^2^) of the standard curve was 0.991, and PCR amplification efficiencies (E) were >0.96, with only one peak in all dissociation curves, thereby demonstrating the specificity and reliability of the analysis. The results indicated a significant reduction in the relative expression level of the viral genome per nanogram of total DNA, by 84 %, 89 % and 73 %, respectively, compared to the mock group at 48 h postinfection, when siRNA704, siRNA1017 and siRNA1388, respectively, were used (Fig. 4).

## Discussion

During RNAi, which is an RNA-mediated pathway of gene silencing mediated by small RNA molecules, introduction of double-stranded RNA (dsRNA) encoding a subsequence of a gene leads to reduction in expression of the corresponding gene in various organisms, including animals and plants [11]. Long double-stranded RNA is cleaved by an endonuclease (Dicer) into 21- to 25-nt fragments (small interfering RNA; siRNA), and these oligonucleotides can serve as guide sequences to direct a multicomponent nuclease to a specific messenger RNA to destroy it [12]. Studies of the antiviral activity of siRNA have shown that it is effective in the inhibition of viral infection and modulation of viral replication in cultured mammalian cells. Examples include HIV-1 [13], severe acute respiratory syndrome virus [14], hepatitis C virus [15], influenza virus [16], swine fever virus (Porntrakulpipat et al., 2010), goatpox virus [9], and Newcastle disease virus [17]. RNAi is increasingly being used to inhibit the replication of viral pathogens and is a promising approach for antiviral therapy in mammals. siRNAs can be introduced into mammalian cells by *in vitro* transcription or by transfection with chemically synthesized siRNAs to achieve effective and very rapid silencing of a target gene, although this approach is limited to cells that can be transfected at high rates, and the effects are transient [8]. The DNA polymerase gene, which is located in the central region of the genome of ORFV, is highly conserved within species and is a highly conserved gene that codes for the most conserved nonstructural protein, which catalyzes the replication of the viral genome and plays a primary role in viral replication [18].

In the present study, the complete genome sequences of the ORFV Jilin isolate was determined. Three pairs of siRNAs were then synthesized based on the complete open reading frame of the DNA polymerase gene of the ORFV Jilin isolate. These siRNAs were then imported into cells by *in vitro* transfection, and the efficiency of inhibition of viral replication was tested using three assays: CPE, TCID_50_ determination and real-time PCR. Our results demonstrate that the three siRNAs were highly capable of inhibiting viral DNA genome replication. Based on the replication kinetics of ORFV in OFTu cells prior to siRNA transfection and the peak period of viral proliferation (data not shown), the cell cultures for viral titration and DNA extraction were harvested at 48 h postinfection.

At 48 h after virus infection, differences in CPE indicated that siRNA704, siRNA1017 and siRNA1388 inhibit virus replication to varying degrees (Fig. 2). To further examine the level of viral inhibition, we tested the viral titers of infected cells by TCID_50_ assay. The results showed that siRNAs caused an approximately 59- to 199-fold decrease in virus yield. Furthermore, the results of real-time PCR indicated that approximately 73-89 % less viral DNA was present in cells transfected with the siRNA than in control cells.

In summary, the use of RNAi directed against the ORFV DNA polymerase gene could effectively inhibit viral replication. Based on the present data and the advantages of siRNA technology, we propose that siRNAs targeting the DNA polymerase gene may be used as a tool to study ORFV replication and pathogenesis in future studies.
